# SERPINC1, a new prognostic predictor of colon cancer, promote colon cancer progression through EMT

**DOI:** 10.1002/cnr2.2079

**Published:** 2024-06-24

**Authors:** Zhenghong Le, Shuran Chen, Yan Feng, Weichen Lu, Mulin Liu

**Affiliations:** ^1^ The First Affiliated Hospital of Jinan University Guangzhou China; ^2^ Department of Gastrointestinal Surgery The First Affiliated Hospital of Bengbu Medical College Bengbu China; ^3^ Department of Gastroenterology Bengbu Third People's Hospital Bengbu China

**Keywords:** biomarkers, colon cancer, epithelial‐mesenchymal transition, liver metastasis, serpin family C member

## Abstract

**Background:**

Liver metastasis of CRC is still the main cause of poor prognosis in patients with CRC. Previous studies have suggested that serpin family C member 1(SERPINC1) is involved in the development of a variety of tumours, but its effect on colorectal cancer progression has been poorly elucidated.

**Methods:**

Based on the GEO database, this study identifies the core gene SERPINC1 associated with liver metastasis in CRC. We used transcriptomic data and immunohistochemical staining to explore the expression of SERPINC1 in normal, cancer, and liver metastases tissue from CRC patients. Clinical data obtained from our hospital were used to explore the impact of SERPINC1 on the prognosis of colon cancer patients. Mechanistically, the biological functions exerted by SERPINC1 in CRC were predicted by bioinformatics, and the results were validated by the results of the experiments in vitro. Cell lines with knockdown of SERPINC1 were performed a series assay such as trans well, CCK‐8 and colony formation assay to explore the relationship between SERPINC1 and proliferation and metastasis of CRC cells. Finally, the effect of SERPINC1 on the sensitivity of colon cancer patients to immune checkpoint therapy was evaluated.

**Results:**

In CRC liver metastatic tissues, we found significantly high expression of SERPINC1. Briefly, 212 CRC cohorts showed that SERPINC1 was significantly associated with TNM stage and plasma CA19‐9 and CEA in CRC patients. Univariate and multivariate Cox demonstrated that SERPINC1 was significantly associated with 5‐year survival after radical surgery for colorectal cancer (*p* < 0.001). Bioinformatics predicted that SERPINC1 affects metastasis of colon cancer through epithelial‐mesenchymal transition (EMT). Colony formation assay and CCK‐8 assay showed that SERPINC1 promotes malignant proliferation of CRC cells, trans well assay showed that SERPINC1 promotes distant migratory behaviour of CRC cells and protein blotting assay showed that SERPINC1 may promote migration by promoting the TGF‐β1‐mediated EMT of CRC cells. In addition, several immunotherapy cohorts also reflected that the expression of SERPINC1 reduced the sensitivity of CRC patients to immune checkpoint therapy.

**Conclusion:**

Our study identified SERPINC1 as a novel liver metastasis‐associated gene in CRC. Targeting SERPINC1 may be a novel therapeutic strategy for patients with liver metastases from CRC.

## 1 INTRODUCTION

Colorectal cancer is one of the leading causes of cancer deaths worldwide.^1^ The detection rate and 5‐year survival rate of early colorectal cancer have been significantly improved with the update of various clinical instruments and the application of multi‐omics, such as radiology and pathology in clinical practise. However, the 5‐year survival rate of advanced colorectal cancer is very low, only about 8%–30%.^2^ In addition to late complications, an important cause of poor prognosis in patients with advanced colon cancer is secondary tumour metastasis, of which liver metastasis accounts for about 70%. Therefore, clarifying the mechanisms of liver metastasis in colorectal cancer is an urgent problem. SERPINC1, a serine protease inhibitor, regulates coagulation stabilisation.^3^ According to previous studies, researchers have detected higher SERPINC1 transcription levels in central nervous system lymphomas. When combined with the patient's prognosis, they concluded that high SERPINC1 transcription levels were associated with poor patient outcomes.^4^ In addition, in some cancers, SERPINC1 promotes tumour migration, invasion, and angiogenesis.^5^ SERPINC1 also has been reported as a potential candidate biomarker for detection of hepatocellular carcinoma.^6^, ^7^

Epithelial‐mesenchymal transition (EMT) is a biological behaviour of tumour cells that has been reported to be associated with malignant proliferation and metastasis for a long time. The essence of this behaviour is the process by which epithelial cells lose their unique epithelial phenotype and remodel the mesenchymal phenotype, which is accompanied by the loss of tight junctions between cells. Several studies have shown that EMT is associated with proliferation and metastasis of colorectal cancer.^8^ A decrease expression of E‐cadherin and an increase expression of vimentin give tumours a high potential for proliferation.^9^ Some key proteins regulating EMT have been found, but the complex molecular mechanisms are still under investigation. Therefore, in this study, we aimed to provide a potential therapeutic target for the treatment of patients with advanced liver metastases from colorectal cancer by analysing the EMT mechanistic impact of SERPINC1 on the regulation of liver metastasis from colorectal cancer.

## 2 MATERIALS AND METHODS

### 2.1 Colorectal cancer patient data acquisition and analysis

This study was based on the GSE81558^10^ and GSE39582^11^ datasets. Adjusted *p* value <.05 and |log2FoldChange| ≥1 were used to screen for differential genes. BEST (https://rookieutopia.com/app_direct/BEST/) was used to analysis the expression between colon cancer and paracancer tissues or colon cancer and colon cancer liver metastasis tissues. Differential oncogenic genes (DEGs) were visualised using “ggVolcano” R package. DAVID (https://david.ncifcrf.gov/home.jsp) was used to perform GO (Gene Ontology) and KEGG (Kyoto Encyclopedia of Genes and Genomes) pathway enrichment analysis. The result of GO and KEGG were visualised using “ggplot” R package. STRING (https://cn.string-db.org/) was used to construct a protein–protein interaction (PPI) network for the selected DEGs. Cytoscape_v3.8.2 was used to visualise PPI networks. The Kaplan–Meier Plotter^12^ was used to analyse the effect of SERPINC1 on the prognosis of patients with colorectal cancer. TIMER^13^ and CellMiner database^14^ were used to analyse the expression of SERPINC1 and its effect on immunotherapy in colon cancer. The CanserSEA single‐cell database was used to analyse the biological functions of SERPINC1 at the single‐cell level.^15^ Finally, BEST website was used to analyse the effect of immune checkpoint inhibitor therapy for patients with colon cancer in SERPINC1‐low and ‐high patients.

### 2.2 Colorectal cancer patients cohort

From February 2016 to December 2017, patients with colorectal cancer who had undergone radical operation and had complete pathological and clinical data were selected. Inclusion criteria: primary colorectal cancer was diagnosed by clinical observation and histopathology, and radical resection was performed successfully. Exclusion criteria: malignant tumour with other tissue origin; malignant neoplasm with other tissue origin; having severe cardiovascular, hepatic or renal disease. According to the above criteria, a total of 212 patients were included and the following information was obtained: (1) basic data: sex, age, preoperative carcinoembryonic antigen (CEA), preoperative carbohydrate antigen 19‐9 (CA 19‐9), tumour diameter, tumour tissue type, pathological grade, T stage, N stage, and so forth. (2) Survival data: the postoperative survival was obtained through telephone follow‐up and outpatient follow‐up. The follow‐up period was up to October 2022, to determine whether and when tumour‐related death occurred 5 years after surgery. This study was approved by the Bengbu Medical College Ethics Committee.

### 2.3 Immunohistochemical staining

The paraffin blocks of tissues were collected from the CRC patients from our hospital. The primary antibody used in the experiment is Anti‐ATIII (1:100, ab126598, Abcam).

### 2.4 Cell culture

Human colon cancer cell (HCT116) and human colonic adenocarcinoma cell (RKO) were all from Wuhan Procell Life Science Co., Ltd. Both cells were cultured in DMEM medium containing 10% fatal bovine serum (GIBCO) and penicillin streptomycin (5000 μ/mL). It was cultured in the incubator with 37°C and 5% CO2.

### 2.5 Construction of knockdown SERPINC1 cell lines

RKO and HCT116 cells were seeded in six‐well plates (3000 cells/well) 24 h before transfection with siRNA, siRNA designed to silence SERPINC1^5^ were obtained from Genepharma (Shanghai, China). siRNA were transfected into HCT116 and RKO cells by using Lipofectamin 6000 transfection reagent (Beyotime, Shanghai, China) according to the manufacturer's instructions.

### 2.6 CCK‐8

Cell proliferation was evaluated using CCK‐8 reagent (Anhui Huaxiao Gene Technogy Co.). Colorectal cancer cells were cultured in 96‐well plates with a number of 1.5 × 103 cells per well. After culturing for 1, 2, 3 and 4 days, respectively, 10‐μL CCK8 solution was added into each well. The absorbance was then measured at 450 nm using a spectrophotometer (Biotek Instruments, Inc., Winooski, VT,) at 37°C for 1.5 h, and the *t*‐test was used to calculate control versus experimental group data.

### 2.7 Cloning experiments

The treated CRC cells were cultured in six‐well plates with 500 cells per well for 14 days. Subsequently, cells were washed with PBS and fixed with methanol for 30 min. Finally, 0.1% crystal violet was stained for 15 min. After the photo was taken, the number and size of clonal plaques were observed.

### 2.8 Transwell experiment

Treated CRC cells were cultured in the upper chamber of a 24‐well plate with a number of 1 × 104 cells per well in serum‐free DMEM medium. Briefly, 500 mL of complete medium was added into the lower chamber. It was cultured in the incubator for 2 days. The upper chamber was fixed with methanol for 30 min. Finally, 0.1% crystal violet was stained for 15 min. After the picture was taken, the number of migrating cells was observed.

### 2.9 Western blotting

The experimental process was referred to the published papers of our research group.^16^ The materials used in the experiment are as follows: (1) RIPA (Beyotime, Shanghai, China); (2) BCA protein assay kit (Beyotime, Shanghai, China); (3) SDS‐PAGE (Epizyme, Shanghai, China); (4) PVDF membrane (Millipore, Billerica, MA, USA) and (5) enhanced chemiluminescence kit (Millipore, Billerica, MA, USA).

### 2.10 Statistic analysis

*T*‐test was used to analyse the significance of differences between groups. Survival analyses were plotted according to Kaplan–Meier curves and log‐rank tests in GraphPad PRISM5. The *p* < .05 was considered statistically significant between the two groups.

## 3 RESULTS

### 3.1 SERPINC1 is associated with liver metastasis from colorectal cancer

**FIGURE 1:**
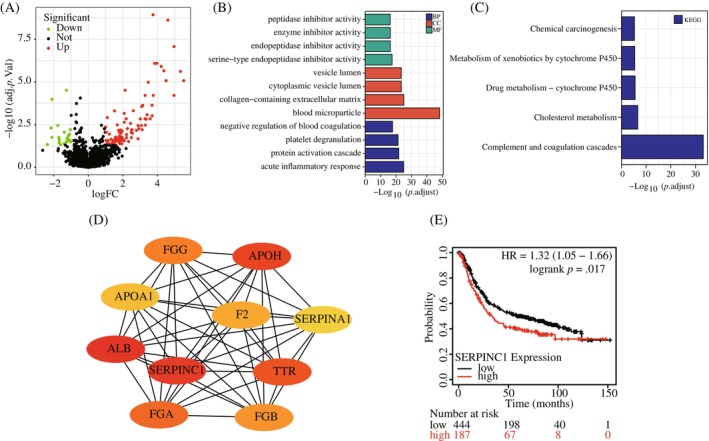
(A) Volcano plot of differentially expressed genes in metastatic versus non‐metastatic tissues; (B, C) Gene Ontology (GO) and Kyoto Encyclopedia of Genes and Genomes (KEGG) enrichment analysis of metastasis‐associated differentially expressed genes; (D) protein–protein interaction network plot of metastasis‐associated core genes and (E) Online database analysis of SERPINC1 expression on colorectal patient prognosis K–M survival curve. SERPINC1, serpin family C member 1.

GSE81558 was analysed by R package, and the DEGs were shown as volcano plots (Figure 1A). Go and KEGG showed that these DEGs were mainly enriched in drug metabolism, platelet degranulation and serine‐type endopeptidase inhibitor activity (Figure 1B,C). The PPI network shows that SERPINC1 is at the core of the differential genes (Figure 1D). The K–M survival curve showed that SERPINC1 was negatively correlated with the survival probability of colon cancer patients (Figure 1E).

### 3.2 SERPINC1 is highly expressed in colorectal cancer

**FIGURE 2:**
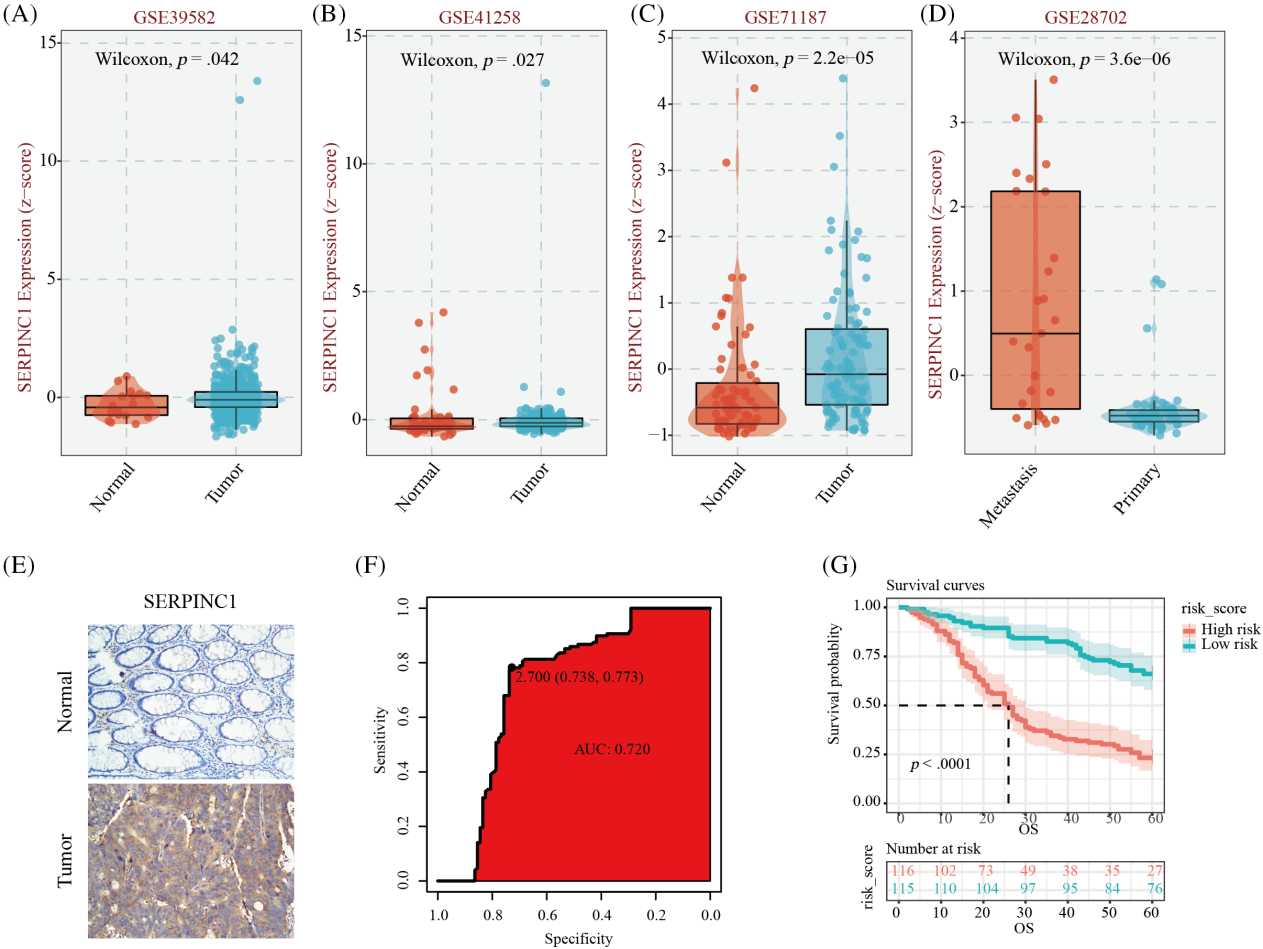
(A–C) The expression of SERPINC1 in colorectal cancer and adjacent normal tissues; (D) relationship between SERPINC1 expression in colorectal cancer metastases and cancer tissues; (E) immunohistochemical staining results of SERPINC1 expression in colorectal cancer and adjacent normal tissues; (F) ROC curve of SERPINC1 in predicting prognosis of colorectal cancer patients and (G) K–M survival curve of the effect of SERPINC1 expression on the survival of colorectal cancer patients. SERPINC1, serpin family C member 1.

Subsequently, we analysed the datasets of GSE39582, GSE41258, GSE71187 and GSE28702. The results showed that the expression of SERPINC1 in colon cancer tissues was significantly higher than that in normal colon tissues (Figure 2A–C), and SERPINC1 was significantly higher in metastatic colon cancer tissues than in non‐metastatic colon cancer tissues (Figure 2D). The results of immunohistochemical staining also indicated that the expression of SERPINC1 was significantly higher in colon cancer tissues than in normal colon tissues (Figure 2E). Subsequently, we performed an analysis of patients with Bengbu Medical College from the first affiliated hospital. The result showing that SERPINC1 had an area under the ROC curve of 0.72 for prognostic prediction of colorectal patients, and had high accuracy in predicting the prognosis of patients with colorectal cancer (Figure 2F). Furthermore, the K–M curve showed that the 5‐year survival of patients with high SERPINC1 expression was lower than that of patients with low SERPINC1 expression (Figure 2E). These results suggest that SERPINC1 is a predictor of poor prognosis in patients with colorectal cancer.

### 3.3 The relationship between SERPINC1 and clinicopathological parameters in colorectal tissues

According to the median value of SERPINC1 expression, 212 colorectal cancer patients were divided into two groups: high SERPINC1 expression group and low SERPINC1 expression group. To investigate the effect of SERPINC1 expression on CEA, CA19‐9, histological grade and TNM stage in peripheral blood of patients with colorectal cancer. The results showed that patients with high SERPINC1 expression had significantly higher levels of CEA and CA19‐9 in peripheral blood, as well as worse T and N stages and a higher proportion of vascular invasion (Table 1).

**TABLE 1:** Relationship between the expression of SERPINC1 protein and clinicopathological parameters in colon cancer.

		*n*	SERPINC1 expression (*n*, %)		*χ* ^2^	*p*
Clinicopathological parameters			Low (*n* = 115)	High (*n* = 116)		
Sex	Male	138	69	69	0.006	.936
	Female	93	46	47		
Age	<60	79	40	39	0.035	.852
	≥60	152	75	77		
Pathological type	Adenocarcinoma	81	42	39	0.075	.784
	others	26	11	15		
CA19‐9 (Ku/L)	<37	131	74	57	5.442	.020
	≥37	100	41	59		
CEA (μg/L)	<5	129	73	56	5.413	.020
	≥5	102	42	60		
Grade	G_1_‐G_2_	73	38	35	0.220	.639
	G_3_	158	77	81		
T Stage	T_1_‐T_2_	126	71	55	4.780	.029
	T_3_‐T_4_	105	44	61		
N Stage	N_0_‐N_1_	117	67	50	5.308	.021
	N_2_‐N_3_	114	48	66		
Vascular invasion	Yes	144	63	81	5.568	.018
	Np	87	52	35		

Abbreviations: CEA, carcinoembryonic antigen; SERPINC1, serpin family C member 1.

### 3.4 The relationship between SERPINC1 expression and clinicopathological parameters in colorectal tissues

Subsequently, we investigated the relationship between SERPINC1 expression and clinicopathological parameters in colorectal tissues. The results showed that in both univariate and multivariate analyses, high SERPINC1 expression was associated with poor 5‐year survival after radical resection for colorectal cancer (Table 2).

**TABLE 2:** Analysis of risk factors affecting 5‐year survival rate after radical colectomy or proctectomy.

Clinicopathological parameters	Univariate Cox		Multivariate Cox	
	Log‐rank *χ* ^2^	*p*	HR (95% CI)	*p*
Sex (male vs. female)	0.850	0.357		
Age (<60 vs. ≥60)	0.147	0.702		
Pathological type (adenocarcinoma vs. others)	0.193	0.661		
CA19‐9 (<37 Ku/L vs. ≥37 Ku/L)	5.826	0.016	1.019 (0.705–1.473)	0.919
CEA level (<5 μg/L vs. ≥5 μg/L)	22.705	<0.001	1.872 (1.290–2.717)	0.001
Grade (G_1_‐G_2_ vs. G_3_)	0.837	0.360		
T Stage (T_1_‐T_2_ vs. T_3_‐T_4_)	17.907	<0.001	1.857 (1.297–2.658)	0.001
N Stage (N_0_‐N_1_ vs. N_2_‐N_3_)	24.001	<0.001	1.725 (1.190–2.499)	0.004
Vascular invasion (Yes vs. No)	11.298	0.001	1.487 (1.005–2.201)	0.047
SERPINC1 (high vs. low)	52.697	<0.001	2.864 (1.930–4.251)	<0.001

Abbreviations: CEA, carcinoembryonic antigen; SERPINC1, serpin family C member 1.

### 3.5 SERPINC1 affects growth of colon cancer cell

**FIGURE 3:**
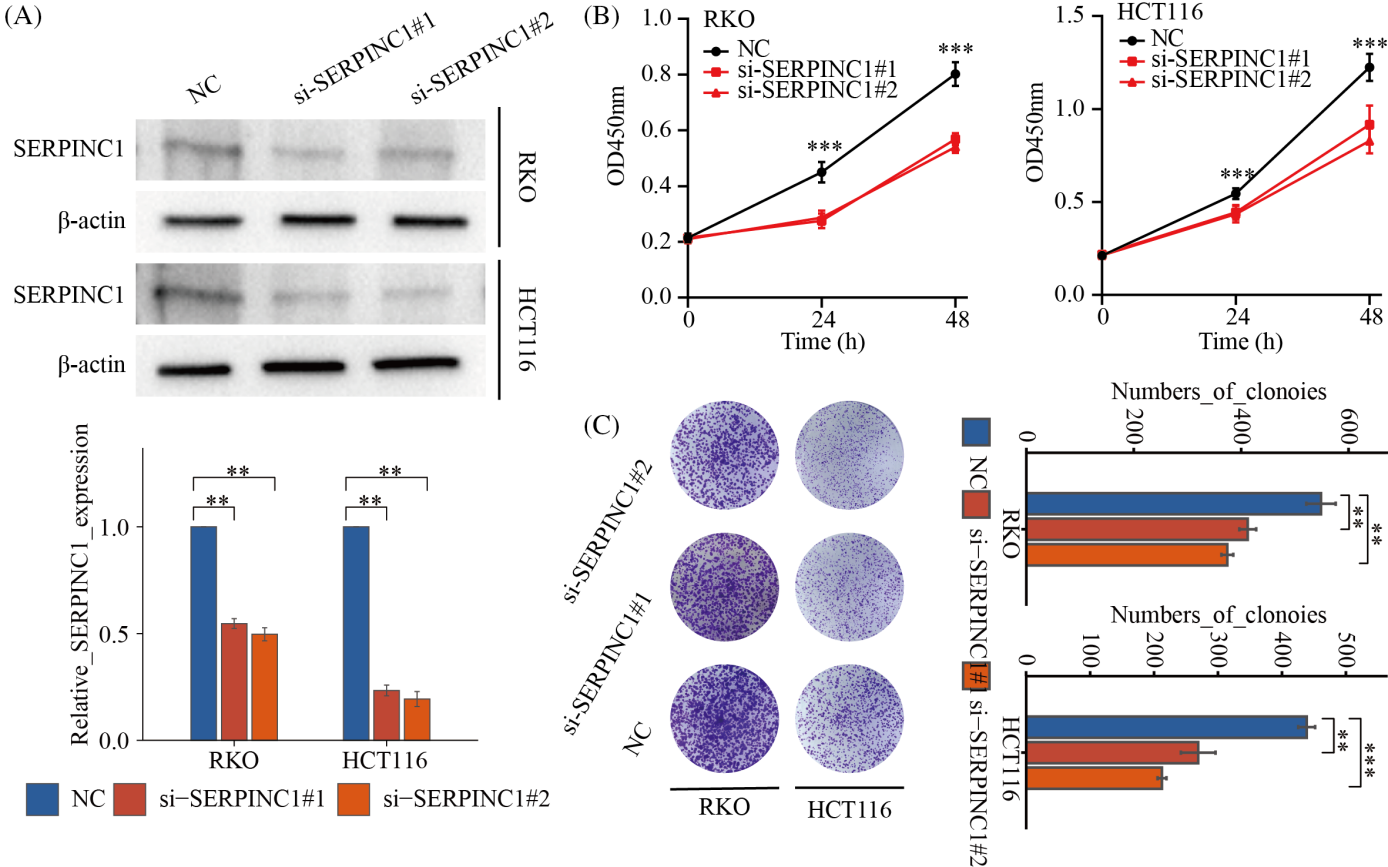
Knockdown of SERPINC1 inhibited the proliferation of colon cancer cells. (A) The expression of SERPINC1 in RKO and HCT116 cells was detected by western blotting. **, *p* < .01. (B) The proliferation of knockdown of SERPINC1 CRC cells was detected by CCK‐8 assay. ***, *p* < .001. (C) The growth of knockdown of SERPINC1 CRC cells was examined by colony formation assay. **, *p* < .01; ***, *p* < .001. SERPINC1, serpin family C member 1.

It has been reported that the high expression of SERPINC1 is associated with the malignant growth of many tumours. We first used western blotting to verify the knockdown efficiency of SERPINC1. The results showed that siRNA using SERPINC1 significantly reduced SERPINC1 expression in both RKO and HCT116 cells (Figure 3A). Subsequently, using the CCK‐8 experiment, we found that knockdown of SERPINC1 significantly reduced the proliferation ability of colon cancer cells (Figure 3B). Finally, clonogenic experiments showed that knockdown of SERPINC1 significantly reduced the monoclonal ability of colon cancer cells (Figure 3C). These results suggest that knockdown of SERPINC1 can inhibit the malignant growth of colon cancer cells.

### 3.6 SERPINC1 affects EMT of colorectal cancer cells

**FIGURE 4:**
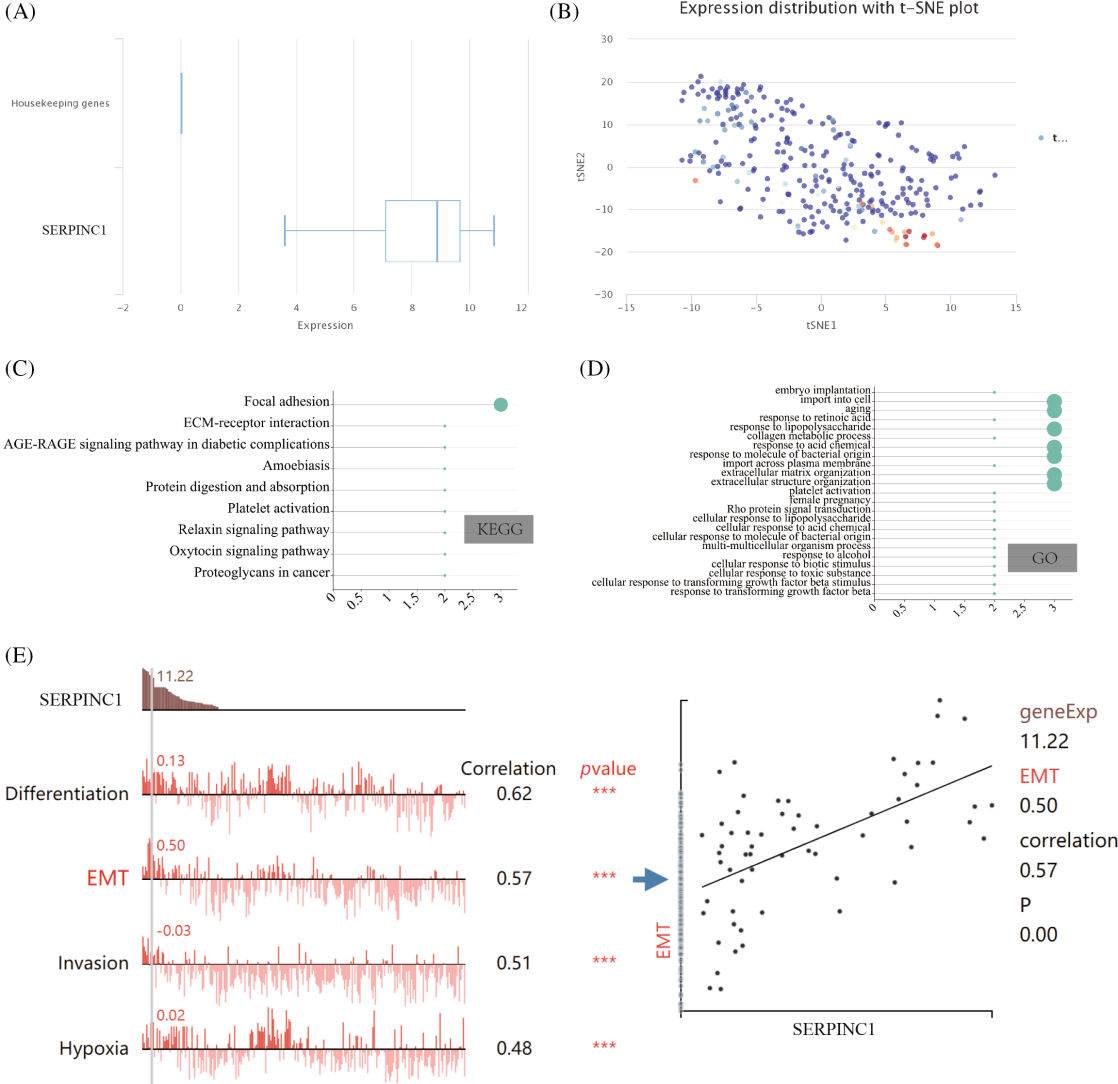
(A, B) Distribution of SERPINC1 in T‐SNE dimensionality reduction plots; (C, D) KEGG and GO enrichment analysis of SERPINC1 and (E) biological functions of SERPINC1 involvement analysed in colorectal cancer single‐cell data. SERPINC1, serpin family C member 1.

To investigate the biological function of SERPINC1 in colorectal cancer, we used bioinformatics to predict its function. First, the expression of SERPINC1 was corrected using housekeeping genes, and subsequent T‐SNE plots revealed significant differences in the expression of different subsets of SERPINC1 in colorectal cancer tissues (Figure 4A,B). Functional enrichment analysis showed that SERPINC1 was closely related to cell metastasis‐related pathways such as focal adhesion and biological functions such as ageing and substance metabolism (Figure 4C,D). Finally, we analysed the function associated with SERPINC1 in single‐cell data and showed that SERPINC1 is closely associated with biological behaviours such as differentiation, EMT and invasion (Figure 4E). Furthermore, based on the expression of SERPINC1, we divided the colon cancer patients in GSE39582 into two groups with high and low expression of SERPINC1 according to the expression of SERPINC1, and performed enrichment analysis for the differential genes between the two groups, SERPINC1 was strongly associated with metastasis of colon cancer cells (additional Figure 1). These results suggest that SERPINC1 may be associated with metastasis of colorectal cancer.

### 3.7 Knockdown of SERPINC1 inhibited TGF β‐induced EMT and migration of colon cancer cells

**FIGURE 5:**
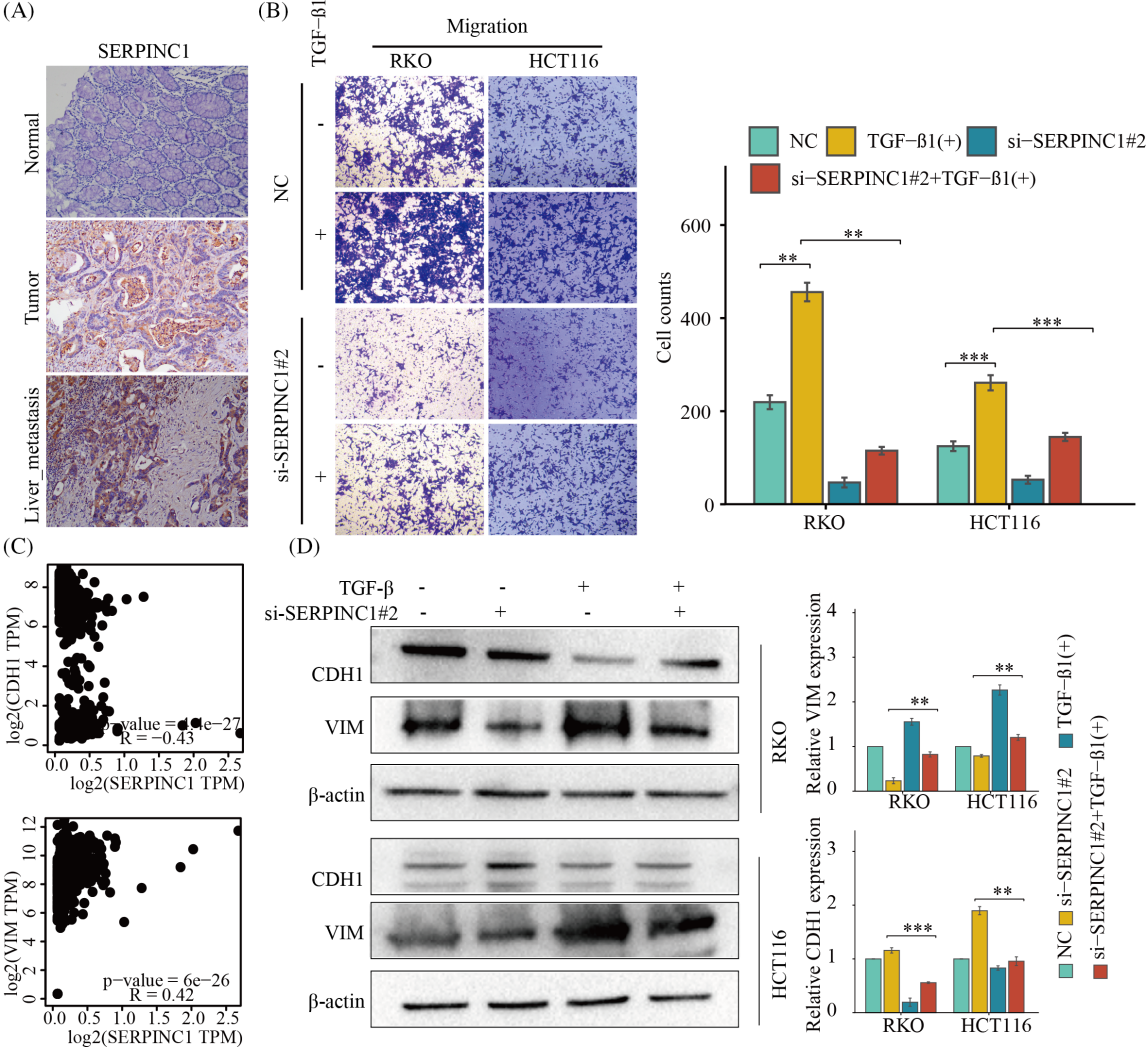
Knockdown of SERPINC1 inhibits TGF‐β mediated colorectal cancer metastasis. (A) The expression of SERPINC1 was detected by immunohistochemistry in normal, cancer and liver metastasis of colon cancer tissue. (B) The migration of RKO and HCT116 cells in knockdown of SERPINC1 CRC cells was examined by trans well assay. **, *p* < .01; ***, *p* < .001. (C) EMT‐related molecules significantly correlated with SERPINC1. (D) VIM and Cdh1 expression in TGF‐β treated and knockdown of SERPINC1 CRC cells was examined by western blotting. **, *p* < .01; ***, *p* < .001. SERPINC1, serpin family C member 1.

To verify that SERPINC1 is indeed associated with liver metastasis of colon cancer, first, we examined SERPINC1 expression in liver metastasis tissues of colon cancer and in situ colon cancer tissues using immunohistochemical staining in 15 colon cancer patients with synchronous liver metastasis. The results showed that the expression of SERPINC1 was significantly increased in liver metastases from colon cancer (Figure 5A). TGF‐β Transforming growth factor beta induces EMT in tumour cells. We first used transwell experiments and found that TGF‐β could significantly promote the migration of colon cancer cells, but this increase in migration capacity could be reversed by knockdown of SERPINC1 (Figure 5B). Given that the previous study found that SERPINC1 was associated with EMT, we screened for EMT core molecules that were significantly associated with SERPINC1: Vim and Cdh1 (Figure 5C). Western blotting showed that TGF‐β could promote the expression of VIM and inhibit the expression of Cdh1 in colon cancer cells. Knockdown of SERPINC1 in colon cancer cells inhibited TGFβ‐induced high expression of VIM, while knockdown of SERPINC1 in colon cancer cells restored TGFβ‐induced reduction of Cdh1 expression (Figure 5D). These results suggest that knockdown of SERPINC1 can inhibit the migration of colorectal cancer, and this process may be related to EMT in colorectal cancer.

### 3.8 SERPINC1 affects the sensitivity of chemotherapy, targeted therapy and immunotherapy in colorectal cancer patients

**FIGURE 6:**
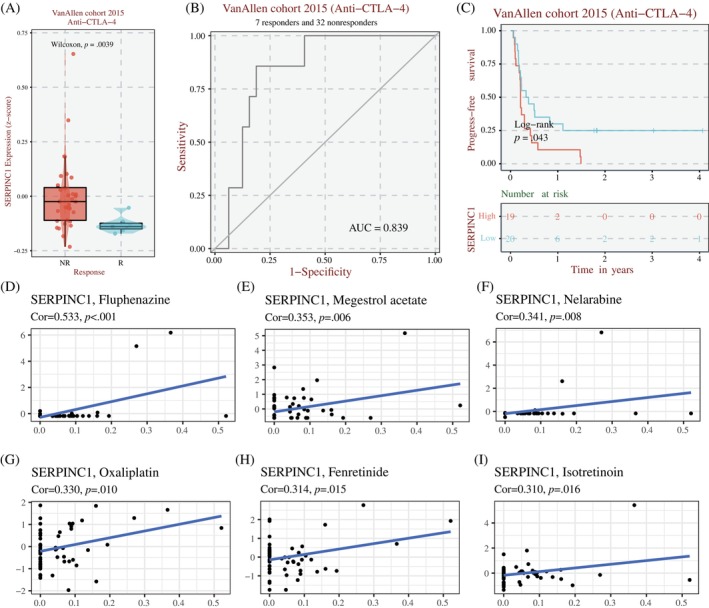
(A) Effect of SERPINC1 expression on the efficacy of patients with colorectal cancer receiving CTLA‐4 inhibitors; (B) ROC curve of SERPINC1 expression predicting the efficacy of patients with colorectal cancer receiving CTLA‐4 inhibitors; (C) K–M survival plot of disease‐free survival of colorectal cancer patients treated with CTLA‐4 inhibitor, and (D–I) correlation analysis of SERPINC1 expression and chemosensitivity of colorectal cancer patients. SERPINC1, serpin family C member 1.

We first used the Timer web site analysis to conclude that SERPINC1 affects the infiltration of multiple immune cells in colon cancer (Figure S2). Subsequently, we performed an immunotherapy‐related analysis of SERPINC1 on the BEST website. In the Van Allen cohort 2015 pairs, we found that high expression of SERPINC1 renders colon cancer patients resistant to treatment with anti‐CTLA‐4 (Figure 6A). Furthermore, this cohort also suggested that colon cancer patients with high expression of SERPINC1 had a lower 5‐year survival after treatment with Ctla‐4 (Figure 6B,C). In addition, when analysing CellMiner data, we also found that high expression of SERPINC1 can contribute to resistance to multiple drug therapies in colon cancer patients, such as oxaliplatin, fenvelamine and nelarabine. These results suggest that SERPINC1 may be associated with resistance to chemotherapy, targeted therapy and immunotherapy in colorectal cancer.

## 4 CONCLUSION

SERPINC1, also known as antithrombin III, is a physiological anticoagulant protein produced by hepatocytes and has been shown to reduce inflammation by inhibiting inflammatory mediators in serum and tissues.^17^, ^18^ SERPINC1 has been shown to regulate the biological behaviour of a variety of malignancies. However, there is no further study to clarify the relationship between Serpinc1 and malignant proliferation and liver metastasis of colorectal cancer.

Analysis of colorectal cancer liver metastasis databases in previous studies has found that SERPINC1 may be associated with colorectal cancer liver metastasis.^19^, ^20^ By analysing the liver metastasis samples of colon cancer in Geo Database, we found that the expression of SERPINC1 in liver metastasis samples was higher than that in colon cancer tissues in situ. Immunohistochemical staining also showed greater expression of SERPINC1 in liver metastases from colon cancer than in normal colon tissues and in situ colon cancer tissues. These results suggest that SERPINC1 may be an important molecule involved in the malignant biological behaviour of colon cancer.

In liver cancer,^21^ previous studies identified SERPINC1 as an important target affecting the prognosis and disease stratification of patients with hepatocellular carcinoma. In nasopharyngeal carcinoma,^5^ the high expression of SERPINC1 was also associated with distant metastasis of NPC. We first analysed on the K–M website that high expression of SERPINC1 contributes to lower 5‐year survival in patients with colon cancer. Subsequently, we explored the association of SERPINC1 with multiple clinicopathologic features in patients with colon cancer by analysing our own cohort of 212 patients with colon cancer. CEA and CA19‐9 are important markers for colon cancer screening.^22^, ^23^ In the results, we first noted that high expression of SERPINC1 was associated with higher CEA and CA19‐9 levels in circulating blood of colon cancer patients. This suggests that the increased expression of SERPINC1 may be related to the pathogenesis of colon cancer. Subsequently, we found that high expression of SERPINC1 was associated with poorer T stage, N stage and deeper vascular invasion in colon cancer patients, these results suggest that SERPINC1 may be an important molecular marker for predicting the prognosis of colon cancer patients.

Liver metastasis of colon cancer is an important factor leading to poor prognosis of patients with colon cancer. In lung cancer,^24^ the researchers demonstrated in vitro and in vivo that overexpression of SERPINC1 promotes the proliferation and distant metastasis of lung cancer cells. In this study, we confirmed that the knockdown of SERPINC1 could significantly inhibit the proliferation and migration of colon cancer cells in vitro. To explore the mechanisms by which Serpinc1 influences various biological behaviours of colon cancer cells, we performed enrichment analysis of its function and pathway using single‐cell and transcriptome data. The results showed that SERPINC1 was closely related to the EMT behaviour in colon cancer. The occurrence of colon cancer and its liver metastasis are closely related to the behaviour of EMT.^25^, ^26^ TGF‐B is a strong inducer of EMT in tumour cells.^27^ Using TGF‐β treated colon cancer cells as a control, we found that knockdown of SERPINC1 inhibited the enhanced migratory ability of colon cancer after TGF‐B treatment. Furthermore, western blot experiments also showed that SERPINC1 regulates the migratory capacity of colon cancer cells by regulating the expression of VIM and Cdh1 in colon cancer cells.

Enhanced EMT of tumour cells is usually associated with resistance to chemoradiotherapy, targeted therapy and immunotherapy.^28^, ^29^, ^30^ Higher SERPINC1 expression is associated with poor tumour microenvironment in lung adenocarcinoma, naive B cells, plasma cells and type M1 macrophages infiltrated more in patients.^31^ In liver cancer, however, studies have shown that higher SERPINC1 expression is inversely associated with M2‐TYPE macrophages that promote tumour progression.^32^ Our analysis of multiple drug sensitivity databases found that high expression of SERPINC1 may contribute to resistance to anti‐CTLA‐4 therapy in patients with colon cancer. The ROC curve and K–M survival curve of treatment also reflected that the benefit of anti‐CTLA‐4 therapy was significantly reduced in patients with high SERPINC1 expression. The high expression of SERPINC1 may also be associated with oxaliplatin resistance in colon cancer. These results suggest that SERPINC1 may play a similar function in colon cancer as it does in lung adenocarcinoma, that is, to inhibit tumour immune process.

In conclusion, we found that SERPINC1 may be closely associated with liver metastasis of colon cancer. This process may depend on SERPINC1 to promote the EMT behaviour of colon cancer cells. Clinical development of novel SERPINC1‐targeted molecular drugs may be helpful in the prevention and treatment of liver metastases from colon cancer.

## AUTHOR CONTRIBUTIONS

**Weichen Lu:** Validation; formal analysis. **Zhenghong Le:** Conceptualization; data curation; validation; formal analysis; writing – original draft. **Shuran Chen:** Writing – original draft; conceptualization; methodology; data curation. **Yan Feng:** Writing – original draft. **Mulin Liu:** Supervision; project administration; writing – review and editing.

## CONFLICT OF INTEREST STATEMENT

The authors declare that they have no financial or personal relationships with other people or organizations that could inappropriately influence their work.

## ETHICS STATEMENT

This study was approved by the Ethics Committee of Bengbu medical university (approval number: 2023AH010068), Patients were consented by an informed consent process that was reviewed by the Ethics Committee of Bengbu medical university and certify that the study was performed in accordance with the ethical standards as laid down in the 1964 Declaration of Helsinki.

## Supporting information


**Figure S1.** SERPINC1 involvement in colorectal cancer metastasis. (A) PCA plot demonstrating that high and low expression of SERPINC1 divides colorectal cancer patients into distinct groups. (B) Volcano plot showing differentially expressed genes between high and low expression groups of SERPINC1. (C) GO enrichment analysis of differentially expressed genes related to SERPINC1.

**Figure S2.** SERPINC1 impacts immunological infiltration in colorectal cancer. (A) The influence of SERPINC1 expression on the infiltration level of different immune cells in colorectal cancer. (B) The impact of varying copy numbers of SERPINC1 on the infiltration level of immune cells in colorectal cancer.

## Data Availability

The data that support the findings of this study are openly available in GEO database at https://www.ncbi.nlm.nih.gov/geo/.
