# Supplementary figures and images for: SERPINC1, a new prognostic predictor of colon cancer, promote colon cancer progression through EMT
Source: Cancer Rep (Hoboken). 2024 Jun 24;7(6):e2079. doi: 10.1002/cnr2.2079 (PMC11194682; doi:10.1002/cnr2.2079)

**A**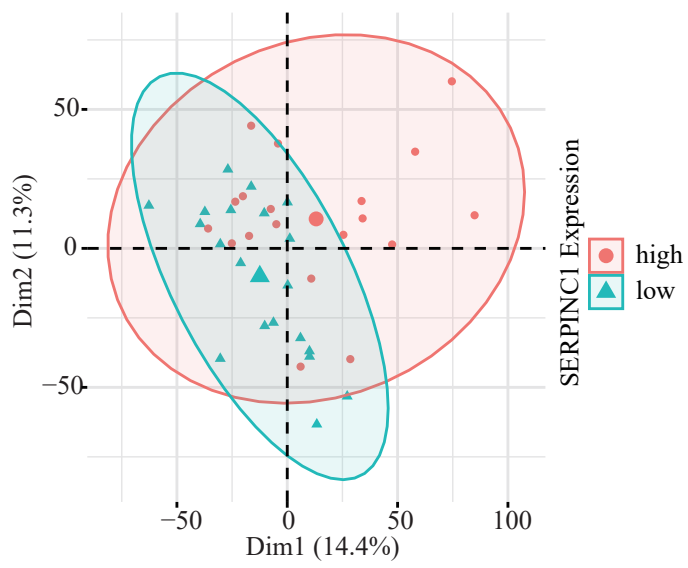**B**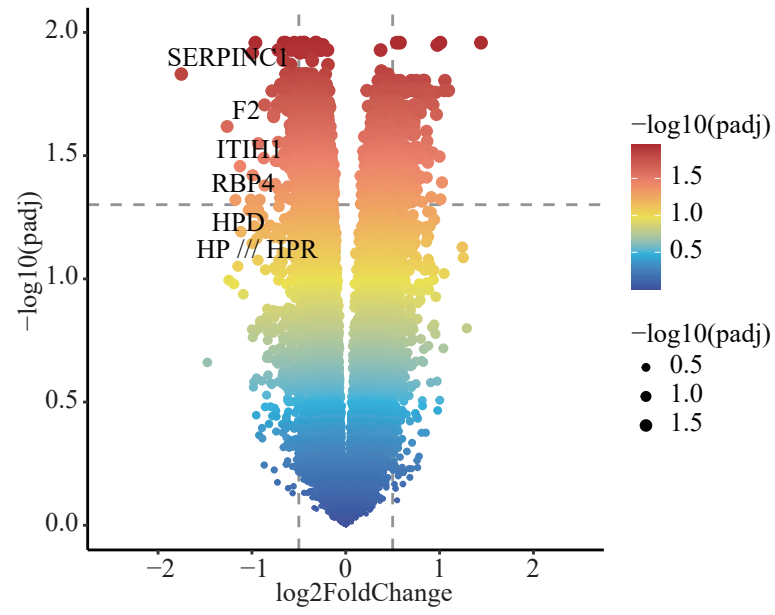**C**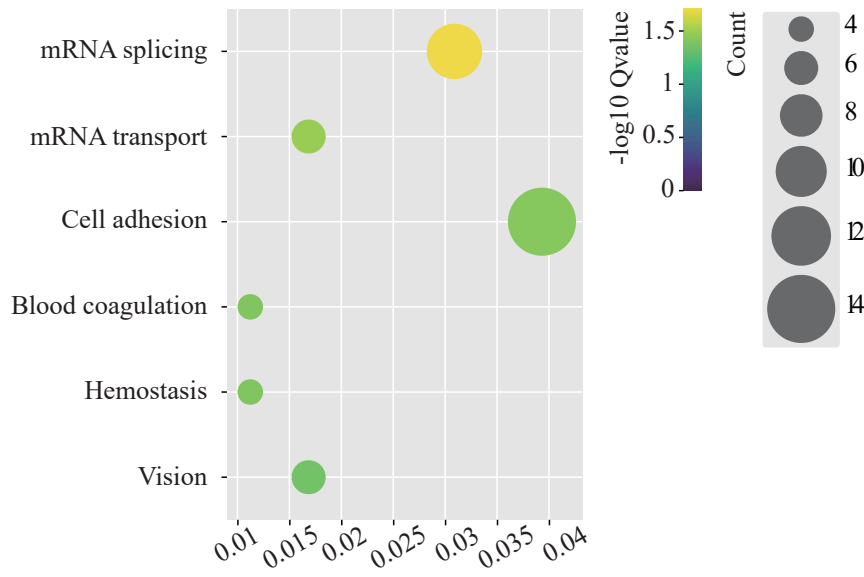

Supplement: Supplementary file 1 — Figure S1. SERPINC1 involvement in colorectal cancer metastasis. (A) PCA plot demonstrating that high and low expression of SERPINC1 divides colorectal cancer patients into distinct groups. (B) Volcano plot showing differentially expressed genes between high and low expression groups of SERPINC1. (C) GO enrichment analysis of differentially expressed genes related to SERPINC1. [file CNR2-7-e2079-s002.pdf]

A

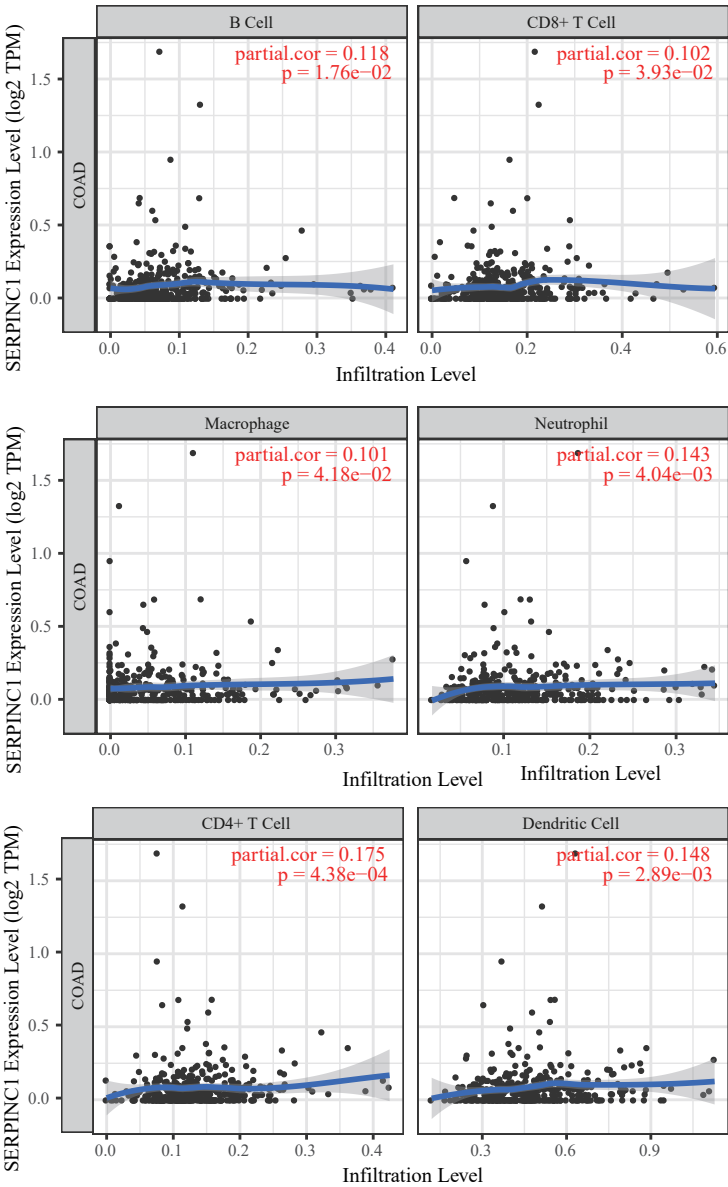

B

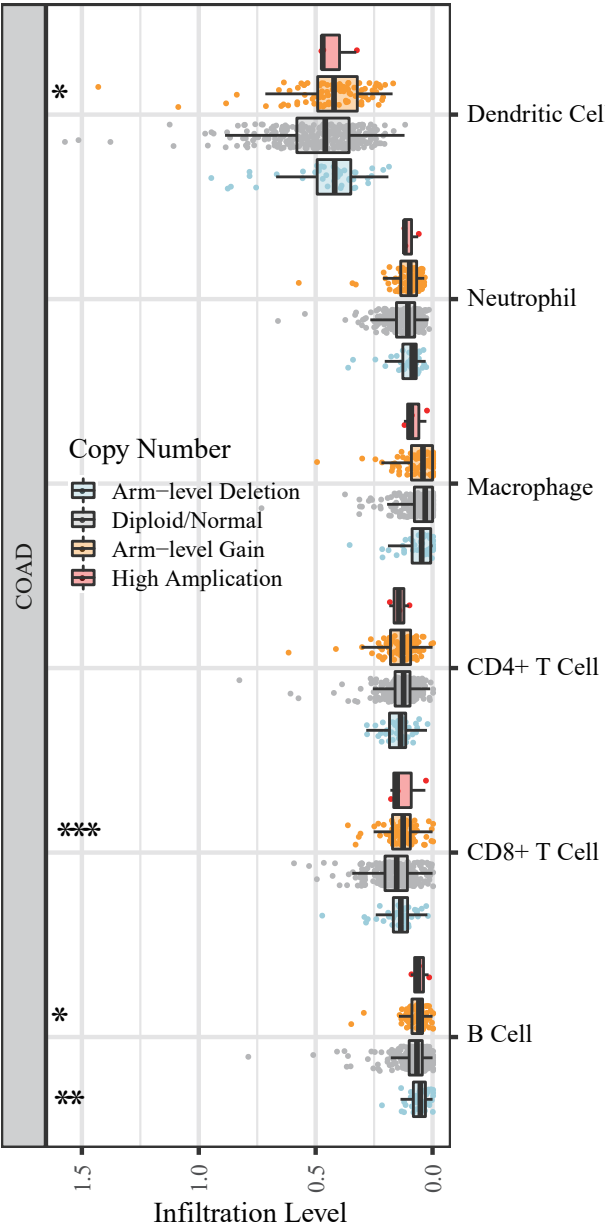

Supplement: Supplementary file 2 — Figure S2. SERPINC1 impacts immunological infiltration in colorectal cancer. (A) The influence of SERPINC1 expression on the infiltration level of different immune cells in colorectal cancer. (B) The impact of varying copy numbers of SERPINC1 on the infiltration level of immune cells in colorectal cancer. [file CNR2-7-e2079-s001.pdf]
